# A Comparison of In Vitro Measurement and Ultrasound for Peripherally Inserted Central Catheter Placement in Premature Infants: A Before-and-After Self-Controlled Prospective Study

**DOI:** 10.7759/cureus.56335

**Published:** 2024-03-17

**Authors:** Shasha He, Jianhui Wang, Xianhong Zhang, Jia Xie, Qingxuan Wan, Ruiyun He, Yanhan Chen, Xuexiu Liu

**Affiliations:** 1 Department of Neonatology, Children’s Hospital of Chongqing Medical University, Chongqing, CHN; 2 College of Nursing, Chongqing Medical University, Chongqing, CHN

**Keywords:** before-and-after self-controlled study, premature infants, picc, ultrasound, in vitro measurement

## Abstract

Background

This study aimed to investigate the effectiveness of ultrasonography (US) and in vitro measurement (IVM) methods in localizing peripherally inserted central catheters (PICCs) in premature infants and analyze the relevant factors affecting the accuracy of IVM.

Methodology

The study employs a prospective before-and-after self-controlled clinical trial design. A total of 210 premature infants who underwent PICC catheterization were compared. We assessed the rate of catheter tip placement, consistency, and stability and analyzed the relevant factors.

Results

The study enrolled a total of 202 premature infants after eight infants dropped out. The one-time positioning rates of the PICC catheter tip using US and IVM were 100% and 73.8%, respectively. Concerning IVM, 53 (26.2%) patients did not reach the optimal position, with 24 (11.8%) patients having a shallow position and 29 (14.3%) having a deep position. The consistency of the two methods was 0.782 (p < 0.05). The degree of dispersion of US was 0.2 (0.0-0.4) cm, which was significantly smaller than IVM at 1.5 (0.0-1.8) cm. Gestational age less than 32 weeks (odds ratio (OR) = 6.64, 95% confidence interval (CI) = 1.43-30.81), weight less than 1,500 g (OR = 5.85, 95% CI = 2.11-16.20), body length less than 40 cm (OR = 15.36, 95% CI = 4.47-52.72), mechanical ventilation (OR = 5.13, 95% CI = 1.77-14.83), abdominal distension (OR = 78.18, 95% CI = 10.62-575.22), and bloating (OR = 8.81, 95% CI = 1.42-47.00) were risk factors that affected the accuracy of IVM.

Conclusions

Gestational age, weight, length, mechanical ventilation, abdominal distension, and swelling can lead to deviations with IVM. US can directly view the tip of the catheter, which is more accurate. Additionally, it is recommended to reduce the length of the catheter by 1.3 cm when using IVM to achieve the best-estimated placement length.

## Introduction

The peripherally inserted central catheter (PICC) is an advanced technique that involves the insertion of a catheter through peripheral veins, meticulously ensuring the catheter tip’s optimal placement within the superior vena cava (SVC) or inferior vena cava (IVC). This methodology provides a secure and consistent infusion route [1]. Notably, PICC is recognized for its safety, durability, and low infection rates as a venous access method, positioning it as a particularly suitable option for newborns, especially premature infants grappling with significant nutritional deficiencies [2]. Given their developmental immaturity, premature infants often require sustained parenteral nutrition, a need effectively met by the PICC technique [3].

Presently, the primary methods utilized for locating the tip of PICC catheters predominantly involve in vitro measurement (IVM), intracavitary electrocardiogram, chest radiography (CR), and ultrasonography (US) [4-7]. IVM stands out as the most frequently employed method in clinical settings, typically determining the catheter tip’s position post-placement via CR [8]. However, the reliability of this method is sometimes questioned, as it may not promptly identify ectopic placements. This limitation can incur additional expenses and unwanted radiation exposure for patients, a concern particularly acute in the case of neonates [9].

In recent years, US has become increasingly widely used in catheter tip positioning due to its advantages of no radiation, real-time, high positioning accuracy, and can make up for the shortcomings of traditional IVM [10]. Research has shown that the PICC catheter in blood vessels under US presents a special imaging structure with a “high low high” sign and directly displays the SVC and IVC and their right atrial entrances, providing a basis for accurately determining the position of the catheter tip inside the vena cava [2].

Therefore, our study aimed to compare the effectiveness of US and IVM in determining the distance between the catheter tip and atrial entrance in premature infants undergoing PICC placement. Furthermore, it aimed to investigate the factors influencing the precision of IVM measurements. To our knowledge, few previous studies have focused on this topic.

## Materials and methods

Estimation of sample size

For estimating the sample size in this study, we utilized the “Confidence Intervals for Kappa” feature within the PASS15 software. Based on clinical experience and preliminary results, the kappa coefficient is estimated to be around 0.776, with a standard deviation of 0.12. Setting the type I error of relevant parameters to 0.05 (α = 0.05) and allowing for a margin of error of 0.05 (δ = 0.05), the calculated sample size is determined to be 91 premature infants. Accounting for a potential 10% loss in the sample, a minimum of 101 premature infants will be required for the study.

Participants

The research conducted at the Department of Neonatology, Children’s Hospital of Chongqing Medical University from September 2022 to March 2023 focused on premature infants requiring PICC catheterization. Inclusion criteria: (1) premature infants requiring PICC catheterization and possessing a suitable physical condition for ultrasound examination; (2) normal bleeding and clotting times; (3) absence of significant peripheral blood vessel constriction or collapse; and (4) parental awareness and signed consent forms. Exclusion criteria: (1) severe congenital heart disease, other cardiovascular conditions, or immunodeficiency disorders; (2) severe intestinal distention hindering catheter tip determination; and (3) instances of transfer, treatment abandonment, or mortality. The study was approved by the Ethics Committee of the Children’s Hospital of Chongqing Medical University (approval number: 2022-381; Clinical trial registration number: ChiCTR2200065371).

Placement of PICC

After placing the neonate in an incubator, using a puncture kit containing a 26 GA (1.9 F) single-lumen PICC catheter, the catheter was inserted according to the neonatal PICC catheterization procedures.

IVM for localization

According to the Practice of Neonatology (5th Edition) [11], the neonates were intubated through the peripheral central vein. For all neonates, the length of the catheter was estimated by IVM and then cut. For the IVM measurement method, the child’s lower limbs were placed in a frog-like position (abduction 45), the segmented length from the corresponding puncture point to the inguinal area to the umbilical region to the sternal xiphoid cartilage was measured, and then added to obtain the total length. For the upper limb, the transverse line of the elbow to the right sternoclavicular joint was measured. The cephalic vein was measured from the pre-puncture point to the third rib. Finally, the obtained length was the estimated tube placement length using the IVM.

The standard for catheter tip positioning

Following the established protocols, it is imperative to avoid placing the catheter tip within the heart of a neonate [12]. The ideal positioning adheres to the 2016 guidelines provided by the American Infusion Nurses Society, which suggest that the safest location for the PICC tip is within the lower third of the SVC or just below the junction of the IVC and right atrium [13].

Locating catheter tips by US

**Figure 1:**
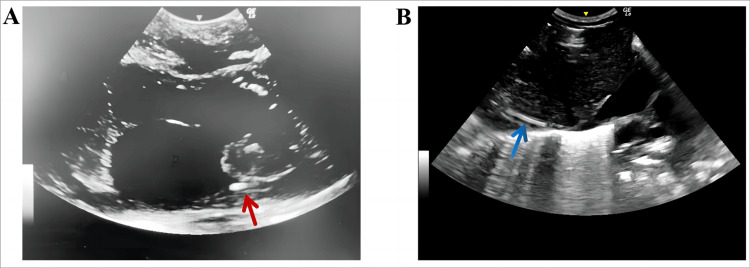
Peripherally inserted central catheter ultrasound imaging of the vena cava. (A) Superior vena cava (red arrow). (B) Inferior vena cava (blue arrow).

Ultrasound procedures were performed utilizing a LOGIQ e color Doppler ultrasonic diagnostic system (6S and 8C probes, GE company, USA) by two skilled PICC specialist nurses with over five years of experience in PICC positioning via ultrasound. The ultrasound probe was strategically positioned either at the midline of the subxiphoid region or along the parasternal line of the right subclavicle region. Subsequently, the probe was rotated clockwise by approximately 15° and tilted rightward to unveil the SVC’s long axis and its entry points into the right atrium (Figure 1A). A small amount of 0.9% sodium chloride solution was infused through the catheter under ultrasound monitoring to confirm the tip’s position, and the distance from the tip to the right atrial inlet was measured. To map the catheter tip in the IVC, the probe was longitudinally positioned along the midsagittal line of the subxiphoid region and scanned along the inferior rib to delineate the IVC and the right atrial inlet (Figure 1B). Following the measurement of the distance from the tip to the atrium, necessary adjustments were made to correct any suboptimal tip placements.

Observation and analysis

One-Time Insertion Rate of the Catheter Tip Using Two Positioning Methods

This is the rate at which the tip of the PICC catheter reaches the optimal position at once. The PICC tip should be located in the lower segment of the SVC, IVC, or near the SVC and right atrium, and should not enter the heart.

Consistency Between US and IVM

After catheterization, US was used to measure and record the distance between the catheter tip and the right atrium. The distance was divided into three intervals, with a value less than 0 cm being 1, 0-2 cm being 2, and greater than 2 cm, being 3. The two sets of data were compared after conversion.

Measurement of Data Volatility Using Two Positioning Methods

The quartile was employed to illustrate the spread of the measured data and compare the variability difference between the two groups of data. A lower data volatility suggests greater accuracy and a more stable, reliable measurement approach.

*Single-Factor and Multiple-Factor Regression Analysis* *Based on the Factors That Affect IVM*

Binary logistic regression analysis was performed for relevant factors such as gestational age, postnatal age, gender, body length, weight at catheterization, catheterization site, times of punctures, mechanical ventilation, abdominal distension, and swelling to identify relevant factors that affected IVM.

Two PICC specialist nurses input all data, and any inconsistencies in results were reviewed and reconciled by both nurses. Subsequently, two individuals entered all information into the computer system, and a third individual verified all electronic data for accuracy.

Statistical analysis

Data analysis was conducted using SPSS version 24.0 software (IBM Corp., Armonk, NY, USA). Normally distributed data were presented as mean ± standard deviation (x ± s), while count data were reported as frequencies and percentages (%). Data variability was expressed using quartiles. Consistency between US and IVM was assessed using kappa coefficient analysis. A p-value <0.05 indicated statistical significance for the differences observed.

## Results

Patients

**Figure 2:**
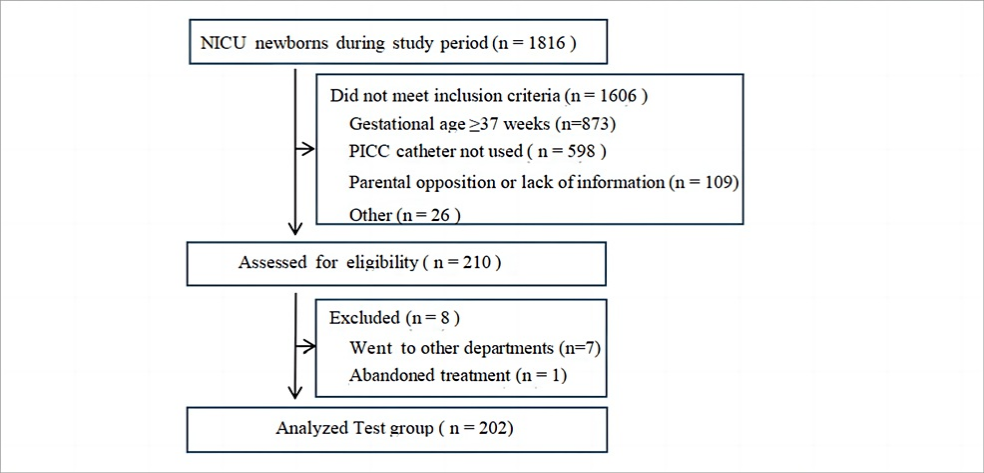
Flowchart of the study population. NICU = neonatal intensive care unit; PICC = peripherally inserted central catheter

Between September 2022 and March 2023, a total of 1,816 neonates underwent screening. Among these, 1,606 were excluded for not meeting the inclusion criteria. Reasons for exclusion included 873 neonates with gestational age ≥37 weeks, 598 instances where a PICC catheter was not utilized, 109 instances where parents of preterm infants declined participation, and 26 cases excluded for other reasons. Initially, 210 premature infants were enrolled, of which eight dropped out (seven transferred to another department and one abandoned treatment). Ultimately, 202 premature infants completed the trials and were included in the final analysis (Figure 2). The number of preterm infants involved in the study met the calculated sample size.

Within the cohort, there were 121 male and 81 female neonates. Among them, 69 required invasive mechanical ventilation, 101 received non-invasive support, and 32 did not undergo mechanical ventilation. Catheterization predominantly involved the lower limbs in 191 neonates, with the great saphenous vein in 128 cases and the superficial femoral vein in 63 cases. Upper limb catheterizations were less common, totaling 11, encompassing one superficial temporal vein, eight basilic veins, and two cephalic veins. The neonates had an average gestational age of 31.75 ± 2.83 weeks. The catheterization was typically performed at 3.09 ± 7.46 days of age, with the neonates weighing an average of 1,758.54 ± 591.58 g at the time of the procedure.

One-time insertion rate of catheter tip using two positioning methods

The one-time positioning rates of PICC catheter tips in neonates using US and IVM were 100% and 73.8%, respectively. Concerning IVM, 53 (26.2%) patients did not reach the optimal position of the catheter tip, with 24 (11.8%) patients having a shallow and 29 (14.3%) patients having a deep PICC catheter tip position, as shown in Table 1.

**Table 1:** One-time insertion rate of the catheter tip between US and IVM at PICC. IVM = in vitro measurement; PICC = peripherally inserted central catheter; US = ultrasound

	PICC tip	Tip position too shallow	Tip position too deep
Cases viewed by US (%)	202 (100)	0	0
Cases viewed by IVM (%)	149 (73.8)	24 (11.8)	29 (14.3)

Consistency between US and IVM

The PICC tip positions measured by US and IVM were compared using SPSS software data conversion, and kappa coefficient analysis was performed to calculate the consistency of the two methods, with a kappa value of 0.782 (p < 0.05) indicating good consistency.

Measurement of data volatility using the two positioning methods

The degree of dispersion of data is represented by the quartile plus coefficient of variation, representing the volatility of the data. Comparing the fluctuation of measurement data between the two positioning methods, the IVM distance was 1.5 (0.0-1.8) cm, with of minimum of -1.0 cm and a maximum of 3.9 cm. The US distance was 0.2 (0.0-0.4) cm, with a minimum of 0.0 cm and a maximum of 1.39 cm. The US distance fluctuation range was small, indicating higher accuracy and higher stability of measurement methods. Non-parametric testing was conducted on the distance difference between US and IVM, and the results showed a statistically significant difference (p < 0.001), with the difference in distance between IVM and US being 1.3 cm.

Factors affecting the accuracy of IVM: single-factor and multiple-factor regression analysis

A binary logistic regression analysis was conducted with accuracy as the dependent variable and 10 factors influencing the accuracy of IVM as independent variables. The variable assignments are shown in Table 2. The results of the single-factor analysis showed that gestational age, postnatal age, gender, body length, weight at catheter placement, catheter insertion site, times of punctures, mechanical ventilation, abdominal distension, and swelling were associated with the accuracy of IVM. For the multiple-factor analysis, variables with a significance level of p < 0.05 in the single-factor analysis were included. Stepwise regression was used for variable selection. The results showed that gestational age less than 32 weeks (odds ratio (OR) = 6.64, 95% confidence interval (CI) = 1.43-30.81), weight less than 1,500 g (OR = 5.85, 95% CI = 2.11-16.20), body length less than 40 cm (OR = 15.36, 95% CI = 4.47-52.72), mechanical ventilation (OR = 5.13, 95% CI = 1.77-14.83), abdominal distension (OR = 78.18, 95% CI = 10.62-575.22), and swelling (OR = 8.81, 95% CI = 1.42-47.00) were identified as risk factors affecting the accuracy of IVM (Table 3).

**Table 2:** Assignment of variables.

Variable	Assignment situation
Gestational age	0 = less than 32 weeks, 1 = greater than 32 weeks
Postnatal age	0 = less than 1 day, 1 = greater than 1 day
Body length	0 = greater than 40 cm, 1 = less than 40 cm
Weight at catheter placement	0 = greater than 1,500 g, 1 = less than 1,500 g
Gender	0 = male, 1 = female
Catheter insertion site	0 = lower limb, 1 = upper limb
Times of puncture	0 = one time, 1 = more than one time
Mechanical ventilation status	0 = no, 1 = yes
Abdominal distension	0 = no, 1 = yes
Swelling	0 = no, 1 = yes

**Table 3:** Single-factor and multiple-factor regression analysis affecting measurement accuracy (n = 202).

Variable	Single-factor regression analysis			Multivariate logistic regression analysis		
	Regression coefficient	P	OR (95% CI)	Regression coefficient	P	OR (95% CI)
Gestational age	0.359	<0.001	0.53 (0.47, 0.60)	1.894	0.015	6.64 (1.43, 30.81)
Postnatal age	0.481	<0.001	0.22 (0.16, 0.28)	0.388	0.588	1.47 (0.36, 6.00)
Body length	0.417	<0.001	0.35 (0.29, 2.42)	2.732	<0.001	15.36 (4.47, 52.72)
Gender	0.397	<0.001	0.39 (0.32, 0.46)	0.131	0.822	0.87 (0.28, 2.74)
Weight at catheter placement	0.394	<0.001	0.40 (0.33, 0.46)	1.767	0.001	5.85 (2.11, 16.20)
Catheter insertion site	0.540	<0.001	0.05 (0.02, 0.09)	1.796	0.125	6.02 (0.60, 9.92)
Times of puncture	0.509	<0.001	0.24 (0.18, 0.30)	18.247	0.998	-
Mechanical ventilation status	0.470	<0.001	0.84 (0.79, 0.89)	1.636	0.003	5.13 (1.77, 14.83)
Abdominal distension	0.535	<0.001	0.08 (0.05, 0.12)	4.359	<0.001	78.18 (10.62, 575.22)
Swelling	0.539	0.001	0.06 (0.03, 0.10)	2.102	0.018	8.81 (1.42, 47.00)

## Discussion

US markedly enhances the accuracy of PICC catheter tip placement, evidenced by a 100% success rate in premature infants, surpassing the 73.8% achieved with IVM. This aligns with previous reports indicating a success range of 85.6% to 100% [14]. Despite advancements, IVM struggles to precisely gauge catheter length due to variables such as patient anatomy, operator technique, and vascular orientation. Neonates, with their pronounced skin folds and variable limb sizes, are particularly prone to measurement discrepancies in IVM [15]. Studies, including ours, reflect this, with 26.2% of cases not achieving optimal placement with IVM, resonating with findings from Wang et al. [16]. IVM’s blind insertion technique, which relies on blood withdrawal for catheter positioning, fails to offer direct visual guidance, leading to imprecise placement and potential post-placement adjustments. These adjustments can escalate treatment costs, infection risks, medical staff workload, and patient radiation exposure [17-19]. The growing demand for precise, non-intrusive, and efficient PICC positioning in clinical settings makes the broad, less accurate approach of IVM increasingly inadequate [20,21]. In contrast, US, with its bedside visualization and precision, addresses these limitations, offering a convenient, repeatable, and accurate method for real-time catheter tip positioning [22,23].

US and IVM demonstrate notable consistency in PICC catheter localization. In our study, a significant agreement between US and IVM was observed (κ = 0.782, p < 0.05), corroborating findings from other studies. For instance, Tauzin et al. [24] employed US in very low birth weight neonates, positioning the probe on the SVC and IVC surface to monitor catheter direction and tip location, thereby enhancing accuracy [25-27]. Diverging from prior research, our analysis delved into the variability of data from both positioning techniques. We noted that US measurements were considerably more stable than those of IVM, with a lesser fluctuation range (0.2 cm vs. 1.5 cm, p < 0.001), underscoring the superior precision and reliability of US. Furthermore, our findings revealed a consistent 1.3 cm discrepancy between IVM measurements and US visuals, suggesting a need to adjust IVM measurements by 1.3 cm for enhanced accuracy.

There is a certain difference between the estimated catheter length based on IVM and the actual ideal position in premature infants. Analyzing factors impacting IVM accuracy revealed that gestational age, weight, body length, mechanical ventilation, and conditions such as abdominal distension and swelling significantly influence measurement precision. The increase in limb size, abdominal distension, and overall swelling in newborns may extend the IVM distance, resonating with this study’s findings on how abdominal distension and swelling affect measurement fidelity [15,27]. Our regression analysis of 202 premature infants identified a correlation between the optimal PICC length and factors such as gestational age, body length, and weight. This aligns with Stroud et al. [28], who, after analyzing data from 727 neonates, formulated an equation for catheter length prediction based on body length and gestational age, mirroring our study’s results. Li [29] further supported this, suggesting a correlation between lower infant weight and shorter vein length. During IVM, operators often face challenges in locating suitable vessels for puncture, complicated by potential vascular malformations in preterm infants, leading to discrepancies between theoretical and actual catheter lengths. Mechanical ventilation also emerges as a notable factor, as it can significantly impact catheter placement and the overall procedure.

Limitations

This study has two limitations that need to be addressed in future research. First, the study’s scope was confined to a single center with a limited sample size. To enhance the robustness of the findings, subsequent research should expand to a multicenter format, encompassing a more substantial participant base. Second, the study lacked a longitudinal dimension, which limits a comprehensive exploration of the advantages and drawbacks of employing US in PICC placement. Future endeavors should incorporate longitudinal studies to provide a more in-depth and temporal understanding of US application in this context.

## Conclusions

This study underscores the notable consistency between US and IVM. It reveals how factors such as gestational age, weight, body length, mechanical ventilation, abdominal distension, and swelling can introduce discrepancies between IVM readings and actual measurements, thereby impacting precision. US stands out for offering direct visualization of the catheter tip, leading to enhanced measurement accuracy. Based on these findings, it is advisable to give precedence to US in clinical settings. Moreover, when employing IVM, adjusting the estimated catheter length by reducing it by 1.3 cm is recommended to ensure more accurate catheter placement.
